# The Dibenzyl Isoquinoline Alkaloid Berbamine Ameliorates Osteoporosis by Inhibiting Bone Resorption

**DOI:** 10.3389/fendo.2022.885507

**Published:** 2022-05-18

**Authors:** Chongjing Zhang, Zeyuan Zhong, Weicong Sang, Farnaz Ghorbani, Behafarid Ghalandari, Marjan Mohamadali, Shiva Irani, Zhi Qian, Chengqing Yi, Baoqing Yu

**Affiliations:** ^1^ Department of Orthopedics, Shanghai Pudong Hospital, Fudan University Pudong Medical Center, Shanghai, China; ^2^ Shanghai Medical College, Fudan University, Shanghai, China; ^3^ State Key Laboratory of Oncogenes and Related Genes, Institute for Personalized Medicine, School of Biomedical Engineering, Shanghai Jiao Tong University, Shanghai, China; ^4^ Department of Biology, Science and Research Branch, Islamic Azad University, Tehran, Iran; ^5^ Institution of Orthopaedic Diseases, Zhangye People’s Hospital Affiliated to Hexi University, Zhangye, China; ^6^ Department of Orthopedics, Shanghai Pudong New Area People’s Hospital, Shanghai, China

**Keywords:** postmenopausal osteoporosis, berbamine, bone marrow-derived macrophage, osteoclast, osteoclastogenesis, DC-STAMP, cell fusion, traditional Chinese medicine

## Abstract

Postmenopausal osteoporosis (PMOP) is a kind of primary osteoporosis that is characterized by decreased bone density and strength. Berbamine is a nonbasic quaternary benzylisoquinoline plant alkaloid that has been widely used in the clinic to treat leukopenia in China. We found that berbamine inhibited RANKL-induced osteoclastogenesis of bone marrow-derived macrophages (BMMs) *in vitro*, which mainly occurred in the middle phase and late phase. The gene and protein expression levels of osteoclast-related molecules, including CTSK, MMP-9, NFATc1, CD44 and DC-STAMP, were also downregulated by berbamine. *In vivo*, we treated PMOP mice with berbamine for 8 weeks and found that the extent of osteoporosis was alleviated significantly according to micro-CT scanning, hematoxylin-eosin staining, DC-STAMP immunohistochemical staining and TRAP immunohistochemical staining in the distal femurs of the mice. Our findings demonstrate that berbamine has an inhibitory effect on the osteoclastogenesis of BMMs and can prevent bone loss after ovariectomy *in vivo*. This study provides evidence that berbamine is a potential drug for the prevention and treatment of PMOP.

## Introduction

Population aging is a global problem that results from combinations of declining rates of fertility and increasing life expectancies. Thus, the proportion of elderly individuals in the total population will increase significantly within the next few decades (1). Primary osteoporosis is a classical age-related disease that increases the risk of fragility fracture (2, 3), and postmenopausal osteoporosis (PMOP) is a common kind of primary osteoporosis (4). PMOP, a chronic and high-bone-remodeling disease that occurs in menopausal women, results in bone microstructure destruction and decreased bone mineral density (BMD) (5).

Osteoclasts are specialized cells that perform bone resorption functions in the human body and they are derived from the monocyte/macrophage hematopoietic lineage. Receptor activator of nuclear factor-kappa B ligand (RANKL) and macrophage colony stimulating factor (M-CSF) are both necessary and sufficient for bone marrow-derived macrophages (BMMs) to undergo the process of osteoclastogenesis and differentiate into osteoclasts (6). M-CSF can facilitate the proliferation, survival and differentiation of osteoclast precursors (7). RANKL leads to the development of mature osteoclasts and it upregulates the expression levels of osteoclast-related genes, such as matrix metalloproteinase 9 (MMP-9), cathepsin K (CTSK) and tartrate-resistant acid phosphatase (TRAP) type 5. RANKL can bind to receptor activator of nuclear factor-kappa B (RANK), which is located on the cell membranes of osteoclast precursors (6, 8).

**Figure 1:**
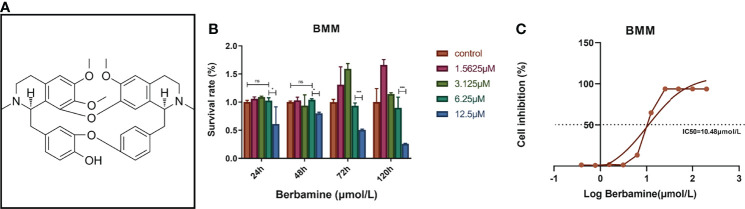
**(A)** The chemical structure of berbamine (https://www.medchemexpress.cn/Berbamine.html). **(B)** Berbamine at concentrations ranging from 0 to 6.25 μM had no significant cytotoxicity toward BMMs at several time points. **(C)** The IC50 of berbamine for BMMs was 10.48 μM. The data are presented as the mean ± SD, *P < 0.05; *** < 0.001; ns, no significance.

Traditional Chinese medicine (TCM) has been used in China for thousands of years. Several studies have reported that various TCM formulas and other Chinese herbal medicines can be used in the prevention and treatment of osteoporosis (9, 10). Our previous studies have reported the potential application value of several TCM monomers in the prevention and treatment of PMOP (11–14). Berbamine is a nonbasic quaternary benzylisoquinoline plant alkaloid that has been proven to have application value in the TCM system (**Figure 1A**) (15). Berbamine has antimicrobial, antineoplastic and anti-inflammatory properties and can allay oxidative stress in HepG2 cells *via* multiple signaling pathways associated with the process of osteoclastogenesis of BMMs (16–18). Thus, we hypothesized that berbamine can attenuate PMOP by inhibiting the osteoclastogenesis of BMMs and the bone resorption function of osteoclasts. We performed this study to investigate the ameliorative effect of berbamine on PMOP and its underlying molecular mechanism.

## Materials and Methods

### Ethics Statement

All animal experiments in this study were approved by the Animal Ethics Committee of Fudan University Pudong Medical Center (No.20211201-1) and were conducted in accordance with the National Institutes of Health (NIH) Guide for the Care and Use of Laboratory Animals.

### Reagents and Antibodies

Berbamine (purity>99%) was purchased from MedChemExpress (New Jersey, USA). We dissolved berbamine in dimethyl sulfoxide (DMSO) to prepare a stock solution (4 mM) and stored the stock solution at -80°C. The stock solution was further diluted with complete culture medium for *in vitro* experiments. For animal experiments, the stock solution was further diluted with DMSO and plant oil. A Cell Counting Kit-8 (CCK-8) was purchased from Dojindo (Tokyo, Japan). A TRAP staining kit was purchased from Sigma–Aldrich (MO, USA). Recombinant m-RANKL and recombinant M-CSF were purchased from R&D Systems (Minneapolis, MN, USA). Alpha-modified minimal essential medium (α-MEM), penicillin–streptomycin (P/S) and fetal bovine serum (FBS) were purchased from Thermo Fisher Scientific (Scoresby, Vic., Australia). Rhodamine-conjugated phalloidin and DAPI were obtained from Solarbio Co., Ltd. (Beijing, China). Universal RNA extraction kits and Evo M-MLV RT kits were purchased from Accurate Biotechnology Co., Ltd. (Hunan, China). Primary antibodies against CTSK, TRAP, MMP-9, NFATc1 (nuclear factor of activated T cells 1), CD44, DC-STAMP (dendritic cell specific transmembrane protein) and GAPDH were purchased from Proteintech (Wuhan, Hubei, China).

### BMMs Isolation and Differentiation

BMMs were isolated and differentiated as described in a previous article (13). We isolated BMMs from the femoral and tibial bone marrow of 6-week-old C57BL/6 mice. We euthanized the mice according to procedures approved by the Animal Ethics Committee of Fudan University Pudong Medical Centre. Sterile complete culture medium (containing 10% FBS and 100 U/ml P/S in α-MEM) was used to flush the femoral medullary cavity, and the BMMs were collected in a sterile dish. Then, the BMMs were cultured in complete culture medium with 20 ng/mL M-CSF for approximately 72 hours. The adherent cells were collected and detected by flow cytometry (BD FACSCaliburTM, USA), and the proportion of BMMs was more than 90% (**Figure S1**). For osteoclast differentiation, BMMs were cultured in complete culture medium containing 50 ng/ml RANKL and 20 ng/ml M-CSF.

### Cell Viability Assay and Determination of the Half-Maximal Inhibitory Concentration of Berbamine

Cell viability was detected by CCK-8 assay according to the kit manufacturer’s protocol. BMMs were cultured in 96-well plates (8×10^3^/well) and incubated with different concentrations of berbamine (12.5, 6.25, 3.125, 1.5625 and 0 μM, 3 duplicate wells for each concentration) for 24, 48, 72 and 120 hours. At each time point, we washed the 96-well plates three times with 1× PBS solution, added 100 μL of CCK-8 working solution to each well, and incubated the plates at 37°C for 1 hour. Then, the absorbance was detected at 450 nm with a microplate reader (Thermo Fisher, Waltham, MA, USA). The half-maximal inhibitory concentration (IC50) of berbamine was detected *via* CCK-8 assay according to the kit manufacturer’s protocol. BMMs were cultured in 96-well plates (8×10^3^/well) and incubated with different concentrations of berbamine (200, 100, 50, 25, 12.5, 6.25, 3.125, 1.5625, 0.78125, 0.390625 and 0 μM, with 3 duplicate wells for each concentration) for 48 hours. We washed the 96-well plates 3 times with 1× PBS solution, added 100 μL of CCK-8 working solution to each well and incubated the plate at 37°C for 1 hour. Then, the absorbance was detected at 450 nm with a microplate reader (Thermo Fisher, Waltham, MA, USA). GraphPad Prism (version 7, GraphPad Software, San Diego, CA, USA) was used to calculate the IC50 of berbamine on BMMs.

### *In Vitro* Osteoclastogenesis Assay

To evaluate the effects of different concentrations of berbamine on the osteoclastogenesis of BMMs, BMMs (8×10^3^/well) were cultured in 96-well plates and divided into a control group (cultured in complete culture medium containing 20 ng/ml M-CSF), a RANKL group (cultured in complete culture medium containing 20 ng/ml M-CSF and 50 ng/ml RANKL) and 2 drug intervention groups (cultured in complete culture medium containing 3.125 or 6.25 μM berbamine, 20 ng/ml M-CSF and 50 ng/ml RANKL).

To evaluate the effects of berbamine (6.25 μM) on BMMs at the different phases of osteoclastogenesis, BMMs (8×10^3^/well) were cultured in 96-well plates and divided into a control group (cultured in complete culture medium containing 20 ng/ml M-CSF), a RANKL group (cultured in complete culture medium containing 20 ng/ml M-CSF and 50 ng/ml RANKL), an early-phase group (cultured in complete culture medium containing 20 ng/ml M-CSF and 50 ng/ml RANKL; berbamine was added to the medium only on days 1-2 of culture), a middle-phase group (cultured in complete culture medium containing 20 ng/ml M-CSF and 50 ng/ml RANKL; berbamine was added to the medium only on days 3-4 of culture) and a late-phase group (cultured in complete culture medium containing 50 ng/ml RANKL and 20 ng/ml M-CSF; and berbamine was added to the medium only on days 5-6 of culture). After 6 days of culture in an incubator with 5% CO2 at 37°C, the BMMs were stained with a TRAP staining kit according to the manufacturer’s protocol. The staining was photographed under a microscope (Nikon Corporation, Tokyo, Japan), and BMMs were regarded as osteoclasts when they contained more than 3 nuclei (11). All experiments were repeated at least 3 times.

### Podosome Belt Formation Assay

To evaluate the effects of different concentrations of berbamine on the osteoclastogenesis of BMMs, BMMs (8×10^3^/well) were cultured in 96-well plates and treated with 3.125 or 6.25 μM berbamine.

To evaluate the effects of berbamine (6.25 μM) on BMMs at the different phases of osteoclastogenesis, BMMs (8×10^3^/well) were cultured in 96-well plates and treated with 6.25 μM berbamine at different phases of osteoclastogenesis. The cells were divided into 5 groups (a control group, a RANKL group, an early-phase group, a middle-phase group and a late-phase group; the details are described above). After 6 days of culture in an incubator with 5% CO2 at 37°C, the cells were fixed with paraformaldehyde (4%) for 20 minutes at 37°C and washed with 1× PBS solution 3 times, and then 0.1% Triton-X 100 was used to increase cell permeability. The cells were incubated in the dark with rhodamine-conjugated phalloidin for 20 minutes and DAPI for 10 minutes. Then, a fluorescence microscope (Nikon Corporation, Tokyo, Japan) was used to detect the podosome belts and nuclei of the cells. BMMs were regarded as osteoclasts when they had more than 3 nuclei (13).

### Pit Resorption Assay

A pit resorption assay was used to evaluate the inhibitory effect of berbamine on the bone resorption function of osteoclasts. BMMs (8×10^3^/well) were plated on the surfaces of bone slices placed in 96-well plates. The cells were divided into 4 groups: a control group (cultured in complete culture medium containing 20 ng/ml M-CSF), a RANKL group (cultured in complete culture medium containing 20 ng/ml M-CSF and 50 ng/ml RANKL) and 2 drug intervention groups (cultured in complete medium containing 3.125 or 6.25 μM berbamine, 20 ng/ml M-CSF and 50 ng/ml RANKL). After 6 days of culture in an incubator with 5% CO2 at 37°C, the bone slices were removed from the 96-well plates and sonicated to remove the cell debris. A scanning electron microscope (GeminiSEM 500, Gemini, Germany) was used to detect the surface topography of the bone slices in the different groups. ImageJ software (NIH, Bethesda, MD, USA) was used to detect the proportion of the pit resorption area on the surfaces of the bone slices in each group.

### Quantitative Real-Time Polymerase Chain Reaction

The expression levels of RANKL-induced osteoclast-related genes, including CTSK, NFATc1, CD44, MMP-9, DC-STAMP and TRAP, were detected *via* quantitative real-time polymerase chain reaction (qRT–PCR). To evaluate the effects of different concentrations of berbamine on the expression levels of RANKL-induced osteoclast-related genes, BMMs (4×10^4^/well) were cultured in 6-well plates and divided into 4 groups (a control group, a RANKL group and 2 drug intervention groups). In the RANKL group, the BMMs were treated with M-CSF (20 ng/ml) and RANKL (50 ng/ml). In the first drug intervention group, the BMMs were treated with M-CSF (20 ng/ml), RANKL (50 ng/ml) and 3.125 μM berbamine. In the second drug intervention group, the BMMs were treated with M-CSF (20 ng/ml), RANKL (50 ng/ml) and 6.25 μM berbamine.

To evaluate the effects of berbamine (6.25 μM) on BMMs at the different phases of osteoclastogenesis, BMMs (4×10^4^/well) were cultured in 6-well plates and treated with 6.25 μM berbamine at different phases of osteoclastogenesis. The cells were divided into 5 groups (a control group, a RANKL group, an early-phase group, a middle-phase group and a late-phase group; the details are described above). After 6 days of culture in an incubator with 5% CO2 at 37°C, the total RNA of the cells was extracted with a Universal RNA Extraction Kit according to the manufacturer’s protocol. Then, the total RNA was reverse-transcribed into cDNA with Evo M-MLV RT kits. The qRT–PCR procedures have been described in detail in our previously published articles. The data was analyzed by the 2^-△△CT^ method. The sequences of the primers are listed in detail in **Table 1**.

**Table 1:** Sequences of the primers used in this study.

Gene	Forward primer	Reverse primer
CTSK	TAGCACCCTTAGTCTTCCGC	CTTGAACACCCACATCCTGC
MMP-9	CGACTTTTGTGGTCTTCCCC	TAGCGGTACAAGTATGCCTCTG
TRAP	TGGGTGACCTGGGATGGATT	AGCCACAAATCTCAGGGTGG
DCSTAMP	GCTGTATCGGCTCATCTCCT	AAGGCAGAATCATGGACGAC
CD44	CCTTGGCCACCACTCCTAAT	TCCGTTCTGAAACCACGTCT
NFATc1	CCAGCTTTCCAGTCCCTTCC	ACTGTAGTGTTCTTCCTCGGC
GAPDH	AGGAGAGTGTTTCCTCGTCC	TGAGGTCAATGAAGGGGTCG

### Western Blotting

Western blotting was performed to evaluate the effects of different concentrations of berbamine on the expression levels of RANKL-induced osteoclast-related proteins, including CTSK, NFATc1, CD44, DC-STAMP, MMP-9 and TRAP, in BMMs. BMMs (4×10^4^/well) were cultured in 6-well plates and divided into 3 groups (a RANKL group and 2 drug intervention groups). In the RANKL group, BMMs were treated with M-CSF (20 ng/ml) and RANKL (50 ng/ml). In the first drug intervention group, BMMs were treated with M-CSF (20 ng/ml), RANKL (50 ng/ml) and 3.125 μM berbamine. In the second drug intervention group, BMMs were treated with M-CSF (20 ng/ml), RANKL (50 ng/ml) and 6.25 μM berbamine.

To evaluate the effects of 6.25 μM berbamine on BMMs at the different phases of osteoclastogenesis *via* Western blotting, BMMs (4×10^4^/well) were cultured in 6-well plates and divided into 5 groups (a control group, a RANKL group, an early-phase group, a middle-phase group and a late-phase group; the details are described above).

To detect whether berbamine can suppress RANKL-induced activation of several common signaling pathways, such as the MAPK, NF-κB and PI3K-AKT-NFATc1 signaling pathways, in the early stage of osteoclastogenesis, we treated BMMs with RANKL (50 ng/ml) and berbamine (6.25 μM) and examined AKT, p65, ERK, IκBα and JNK phosphorylation at four time points (0, 15, 30, and 60 minutes).

The treated BMMs were lysed on ice in radioimmunoprecipitation assay lysis buffer (Teye, Bioteke Corp., Beijing, China). Sodium dodecyl sulfate–polyacrylamide gel electrophoresis (SDS–PAGE) was used to separate equal amounts of osteoclast-related proteins, and the separated proteins were transferred to polyvinylidene fluoride (PVDF) membranes (GE Healthcare, Silverwater, Australia). The membranes were blocked with 5% fat-free milk solution for 60 minutes at room temperature and then incubated at 4°C overnight with primary antibodies against CTSK, NFATc1, CD44, MMP-9, DC-STAMP, TRAP and GAPDH. Subsequently, the membranes were incubated with corresponding secondary antibodies for 60 minutes at room temperature. The protein bands were visualized with enhanced chemiluminescence (ECL) liquid (Teye, Bioteke Cor. Beijing, China) and an image development device (Amersham Pharmacia Biotech, Sydney, NSW, Australia).

### Molecular Docking of Berbamine With DC-STAMP

We simulated the molecular docking of berbamine with DC-STAMP *via* AutoDock 4 software (version 4.2.6, Scripps Research Institute, La Jolla, CA, USA). The PDB-format file of the three-dimensional (3D) structure of DC-STAMP was downloaded from UniProt (https://www.uniprot.org/; UniProt ID of DC-STAMP: Q7TNJ0). The 2D structure of berbamine was sketched in ChemDraw (version 20.0, CambridgeSoft, USA), converted to a 3D structure *via* the Chem3D Ultra program (version 10.0, CambridgeSoft, USA) and saved as a PDB-format file. The charges were calculated, and hydrogen atoms were added to the 3D structures of both DC-STAMP and berbamine with AutoDockTools (version 1.5.7, Scripps Research Institute, La Jolla, CA, USA) to minimize the system’s energy. After completing the above steps, the binding pocket of DC-STAMP and berbamine was predicted *via* sitemap, and then molecular docking of DC-STAMP and berbamine was performed using AutoDock 4 software (version 4.2.6, Scripps Research Institute, La Jolla, CA, USA).

### Animal Experiments

The animal experiments were conducted in a specific pathogen-free (SPF) animal laboratory. Female six-week-old C57BL/6 mice were purchased from Shanghai Laboratory Animal Research Centre (Shanghai, China). All mice were housed in an SPF animal laboratory with clean water and fodder. All mice were randomly divided into 3 groups [a sham group, an ovariectomy (OVX) group and a berbamine group, n=5]. The mice were intraperitoneally injected with 2% (w/v) pentobarbital (40 mg/kg) for anesthesia. The mice in both the OVX group and the berbamine group underwent bilateral OVX *via* a dorsal approach, while the mice in the sham group only underwent removal of a small volume of fatty tissue from around their ovaries *via* the same approach. After the incisions were properly sutured and the animals recovered from anesthesia, the mice were returned to their own cages and allowed to recover for 3 days before intraperitoneal injection. The mice in the berbamine group received berbamine (20 mg/kg) dissolved in DMSO (CDMSO<0.2%) and plant oil every two days for eight weeks. The mice in both the sham group and the OVX group received the same solvent only through intraperitoneal administration every two days for eight weeks. Calcein (10 μg/g) was injected into the mice 10 days and 3 days before the animals were sacrificed *via* intraperitoneal injection. After eight weeks, all mice were euthanized, and the bilateral femurs and serum of the mice were collected for the follow-up experiments.

### Micro-Computed Tomography Analysis of Distal Femur of Mice

The right femurs of the mice were scanned by microcomputed tomography (Skyscan1172, Bruker, Kontich, Belgium). The tube voltage was set to 50 kV with an electric current of 500 μA, and the voxel size of the reconstructed image was 9×9×9 μm³. We analyzed the images for the following histological parameters: the BMD, trabecular number (Tb.N), trabecular pattern factor (Tb.Pf), bone surface (BS)/bone volume (BV) ratio, BS density (BS/total volume [TV]), and percent BV (BV/TV). We reconstructed two-dimensional (2D) and 3D images of the distal femurs of the mice with Mimics 18.0 software (Materialise, Leuven, Belgium).

### Bone Histological Analysis of Distal Femur of Mice

We fixed the right femurs of the mice in 4% paraformaldehyde at room temperature. A Leica SM2500 microtome (Germany) was used to prepare unstained 3-μm-thick sections, and a fluorescence microscope was used to detect the calcein labels in the distal femurs at 515 nm.

We fixed the left femurs of the mice and decalcified them in 10% tetrasodium-EDTA for 14 days. Then, the left femurs were paraffin-embedded, and 4-μm-thick sections were prepared with a microtome (Jung, Heidelberg, Germany). We used hematoxylin and eosin to stain trabecular bone. TRAP immunohistochemical staining was performed to detect TRAP-positive osteoclasts in the distal femurs of the mice in each group. DC-STAMP immunohistochemical staining was performed to detect DC-STAMP-positive osteoclasts in the distal femurs of the mice in each group. The mineral apposition rate (MAR) was used to measure osteoblastic activity by measuring the average distance between two labels. All data were calculated with ImageJ software (NIH, Bethesda, MD, USA).

### Statistical Analysis

All experiments were repeated 3 times, and the results are presented as the $\bar{\chi }$ ± SD for data that were normally distributed. In this study, GraphPad Prism (version 7, GraphPad Software, San Diego, USA) was used for statistical analysis. Comparisons between two groups were performed by Student’s t test for normally distributed variables or Mann–Whitney U test for nonnormally distributed variables. One-way ANOVA with Tukey’s *post-hoc* test was used to determine whether the differences among several groups were statistically significant. Values of P<0.05 were considered to indicate statistically significant differences.

## Results

### Effect of Berbamine on BMM Viability

BMMs were treated with different concentrations of berbamine (0, 1.5625, 3.125, 6.25 and 12.5 μM), and the absorbance at 450 nm was tested after 24, 48, 72 and 120 hours of treatment to determine the cytotoxicity of berbamine. The data revealed that berbamine had no significant cytotoxicity toward BMMs at concentrations less than or equal to 6.25 μM (**Figure 1B**). BMMs were also cultured in 96-well plates (8×10^3^/well) and incubated with different concentrations of berbamine (200, 100, 50, 25, 12, 5, 6.25, 3.125, 1.5625, 0.78125, 0.390625 and 0 μM) for 48 hours. The IC50 of berbamine for BMMs was calculated to be 10.48 μM (**Figure 1C**).

### Berbamine Mainly Inhibits RANKL-Induced Osteoclastogenesis of BMMs *In Vitro* in the Middle and Late Phases

**Figure 2:**
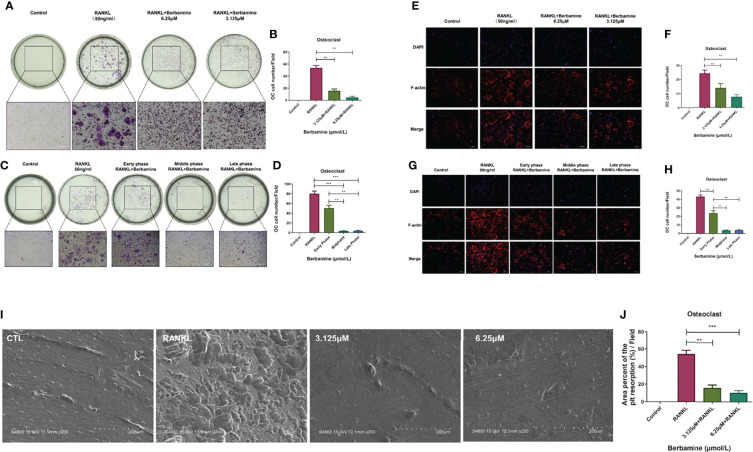
Berbamine can mainly inhibit RANKL-induced osteoclastogenesis of BMMs *in vitro* in the middle and late phases. **(A)** The results of TRAP staining revealed that berbamine (3.125 and 6.25 μM) could inhibit RANKL-induced osteoclastogenesis of BMMs *in vitro*. **(B)** The numbers of mature, polynuclear osteoclasts were reduced significantly in a dose-dependent manner after treatment with berbamine. **(C)** The results of TRAP staining revealed that berbamine inhibited osteoclastogenesis of BMMs mainly in the middle and late phases, not in the early phase. **(D)** The numbers of mature, polynuclear osteoclasts were reduced significantly when berbamine treatment was conducted in the middle phase or late phase of osteoclastogenesis. **(E)** Berbamine (3.125 and 6.25 μM) inhibited RANKL-induced podosome belt formation of BMMs *in vitro*. **(F)** The numbers of mature, polynuclear osteoclasts were reduced in a dose-dependent manner when BMMs were treated with berbamine. **(G)** Berbamine inhibited RANKL-induced podosome belt formation of BMMs mainly in the middle and late phases of osteoclastogenesis, not in the early phase. **(H)** The numbers of mature, polynuclear osteoclasts were reduced in a dose-dependent manner when BMMs were treated with berbamine. **(I)** Masses of bone resorption pits were observed on the surfaces of the bone slices in the RANKL group. **(J)** The percent area of pit resorption on the surfaces of bone slices was significantly higher in the RANKL group than in the berbamine treatment groups. The data are presented as the mean ± SD, **P < 0.01; ***P < 0.001.

To determine the effect of berbamine on the osteoclastogenesis of BMMs, we plated BMMs into 96-well plates and divided them into four groups. We observed that the cells in the two drug intervention groups were dyed by the TRAP staining kit. However, the results revealed that berbamine (both 3.125 μM and 6.25 μM) inhibited RANKL-induced osteoclastogenesis of BMMs *in vitro* compared with that in the RANKL group. We found that the numbers of mature, polynuclear osteoclasts were reduced significantly in a dose-dependent manner after treatment with berbamine (**Figures 2A, B**).

The progression of osteoclastogenesis consists of multiple phases. Thus, it was important for us to determine the phase in which berbamine could inhibit RANKL-induced osteoclastogenesis of BMMs. BMMs were plated into 96-well plates and treated with berbamine (6.25 μM) at different phases of osteoclastogenesis. The results revealed that the inhibitory effect of berbamine on osteoclastogenesis of BMMs mainly occurred in the middle and late phases. However, the inhibitory effect was relatively weak in the early phase (**Figures 2C, D**).

### Berbamine Can Inhibit RANKL-Induced Podosome Belt Formation *In Vitro* in the Middle and Late Phases of Osteoclastogenesis

We plated BMMs into 96-well plates and treated them with berbamine to determine the inhibitory effect of berbamine on RANKL-induced podosome belt formation during the progression of osteoclastogenesis. Berbamine (both 3.125 μM and 6.25 μM) inhibited RANKL-induced podosome belt formation of BMMs *in vitro*, and the numbers of osteoclasts were reduced in a dose-dependent manner after treatment with berbamine (**Figures 2E, F**).

Similar to previous results, the results revealed that berbamine inhibited RANKL-induced podosome belt formation in BMMs mainly in the middle and late phases of osteoclastogenesis. However, the inhibitory effect of berbamine was relatively weak in the early phase of osteoclastogenesis (**Figures 2G, H**).

### Berbamine Can Inhibit RANKL-Induced Formation of Osteoclasts With Bone Resorption Function *In Vitro*

We placed bone slices into 96-well plates, plated BMMs on the surfaces of the bone slices and divided them into 4 groups (a control group, a RANKL group and 2 drug intervention groups) to determine the inhibitory effect of berbamine on RANKL-induced formation of osteoclasts with bone resorption function *in vitro*. After 6 days, the bone slices were fixed with paraformaldehyde (4%) and sonicated to remove the cell debris from the surfaces. Then, the surface morphology of the bone slices was investigated by scanning electron microscopy (**Figure 2I**), and the data showed that the percent area of pit resorption on the surfaces of bone slices was significantly higher in the RANKL group than in the berbamine treatment groups (**Figure 2J**), which revealed that berbamine (both 3.125 μM and 6.25 μM) could inhibit RANKL-induced formation of osteoclasts with bone resorption function *in vitro*.

### Berbamine Can Downregulate the Expression Levels of RANKL-Induced Osteoclast-Related Genes *In Vitro* in the Middle and Late Phases

**Figure 3:**
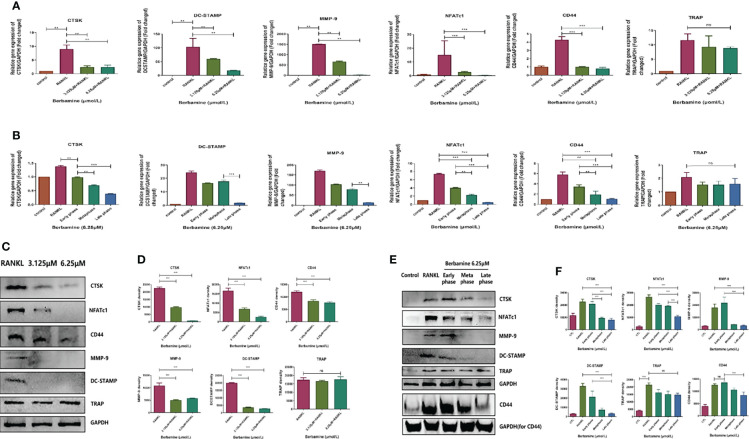
**(A)** qRT–PCR was performed to determine the inhibitory effects of berbamine (both 3.125 μM and 6.25 μM) on the expression of RANKL-induced osteoclast-related genes (CTSK, DC-STAMP, MMP-9, NFATc1, CD44 and TRAP). The expression levels of these genes were normalized to those of GAPDH. **(B)** qRT–PCR was performed to determine the inhibitory effects of berbamine (6.25 μM) on the expression of RANKL-induced osteoclast-related genes (CTSK, DC-STAMP, MMP-9, NFATc1, CD44 and TRAP) at different phases of osteoclastogenesis. The expression levels of these genes were normalized to those of GAPDH. **(C)** Western-blotting was performed to determine the inhibitory effect of Berbamine (both 3.125μM and 6.25μM) on the protein expression level of CTSK, NFATc1, CD44, MMP-9, DC-STAMP and TRAP. **(D)** Quantification of CTSK, NFATc1, CD44, MMP-9, DC-STAMP and TRAP expression levels. **(E)** Western blotting was performed to determine the effects of berbamine (6.25 μM) on the protein expression levels of CTSK, NFATc1, CD44, MMP-9, DC-STAMP and TRAP at different phases of osteoclastogenesis. **(F)** Quantification of CTSK, NFATc1, CD44, MMP-9, DC-STAMP and TRAP expression levels at different phases of osteoclastogenesis. The data are presented as the mean ± SD, **P < 0.01; ***P < 0.001; ns, no significance.

We used qRT–PCR analysis to determine the inhibitory effects of berbamine on the expression of RANKL-induced osteoclast-related genes, such as CTSK, DC-STAMP, MMP-9, NFATc1, CD44 and TRAP. BMMs were plated in 6-well plates and treated with berbamine (0, 3.125, and 6.25 μM). Berbamine (both 3.125 μM and 6.25 μM) significantly downregulated the expression levels of the osteoclast-related genes CTSK, DC-STAMP, MMP-9, NFATc1 and CD44; however, it did not inhibit the gene expression of TRAP (**Figure 3A**). BMMs were also plated into 6-well plates and treated with berbamine (6.25 μM) at different phases of osteoclastogenesis. The results revealed that berbamine significantly downregulated the expression levels of the osteoclastogenic genes CTSK, DC-STAMP, MMP-9, NFATc1 and CD44 mainly in the middle and late phases of osteoclastogenesis. However, treatment of BMMs with berbamine did not significantly downregulate the expression level of the TRAP gene (**Figure 3B**).

### Berbamine Can Inhibit RANKL-Induced Osteoclast-Related Protein Expression

Similar to the previous qRT–PCR results, the protein expression levels of CTSK, NFATc1, CD44, MMP-9 and DC-STAMP were downregulated by berbamine (both 3.125 μM and 6.25 μM) in a dose-dependent manner. However, TRAP protein expression was not significantly downregulated by berbamine (**Figures 3C, D**). Berbamine significantly downregulated CTSK, NFATc1, CD44, MMP-9 and DC-STAMP protein expression mainly in the middle and late phases of osteoclastogenesis. However, it did not significantly downregulate the protein expression level of TRAP (**Figures 3E, F**).

### Berbamine Cannot Inhibit RANKL-Induced Activation of Multiple Common Signaling Pathways in the Early Phase of Osteoclastogenesis

**Figure 4:**
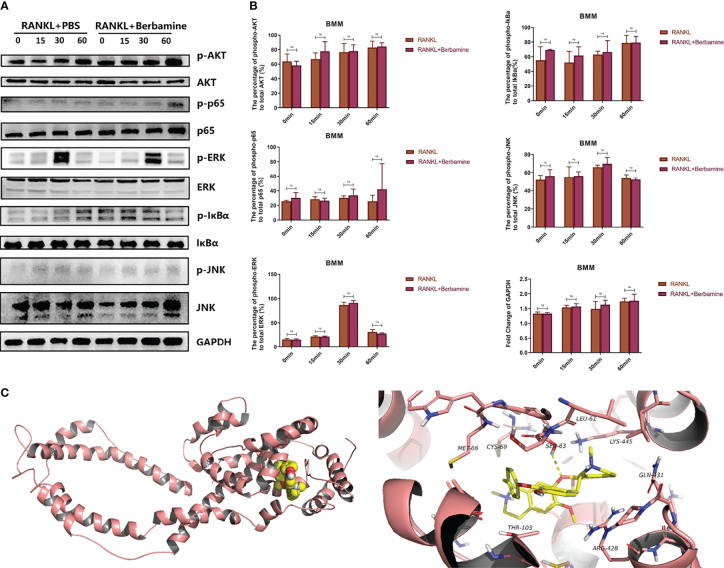
**(A)** Berbamine cannot inhibit RANKL-induced activation of multiple common signaling pathways in the early phase of osteoclastogenesis, western blot images showing the phosphorylated and total protein levels of AKT, p65, ERK, IκBα and JNK induced by RANKL with or without berbamine. **(B)** Quantification of phosphorylated and total AKT, p65, ERK, IκBα, and JNK protein expression levels. The data are presented as the mean ± SD; ns, no significance. **(C)** Molecular docking of berbamine with DC-STAMP. The pink ribbons represent DC-STAMP, and the 3D structure that consists of yellow sticks represents berbamine. The yellow dashed lines represent H-bonds. The calculated free energy of the binding of berbamine with DC-STAMP is -5.808 kcal/mol.

RANKL induced the activation of multiple common signaling pathways, such as the MAPK signaling pathway, the NF-κB signaling pathway and the PI3K-AKT-NFATc1 signaling pathway, which play important roles in the process of osteoclastogenesis. We used Western blot analysis to examine the levels of phosphorylated AKT, p65, ERK, IκBα and JNK in order to detect whether berbamine could inhibit RANKL-induced activation of the corresponding common signaling pathways in the early phase of osteoclastogenesis and found that it could not (**Figures 4A, B**).

### Molecular Docking of Berbamine With DC-STAMP

We used AutoDock 4 to simulate the molecular docking of berbamine with DC-STAMP. The results predicted that berbamine can bind with DC-STAMP by forming a stable hydrophobic interaction with a binding pocket consisting of MET-66, CYS-69, LEU-61, LYS-445, GLN-431, ARG-428 and THR-103. In addition, berbamine can form hydrogen bonds with SER-63 of DC-STAMP. The free energy of the binding of berbamine with DC-STAMP was calculated to be -5.808 kcal/mol; thus, berbamine can bind tightly to DC-STAMP (**Figure 4C**).

### Berbamine Can Prevent Bone Loss by Inhibiting the Bone Resorption Function of Osteoclasts *In Vivo*

**Figure 5:**
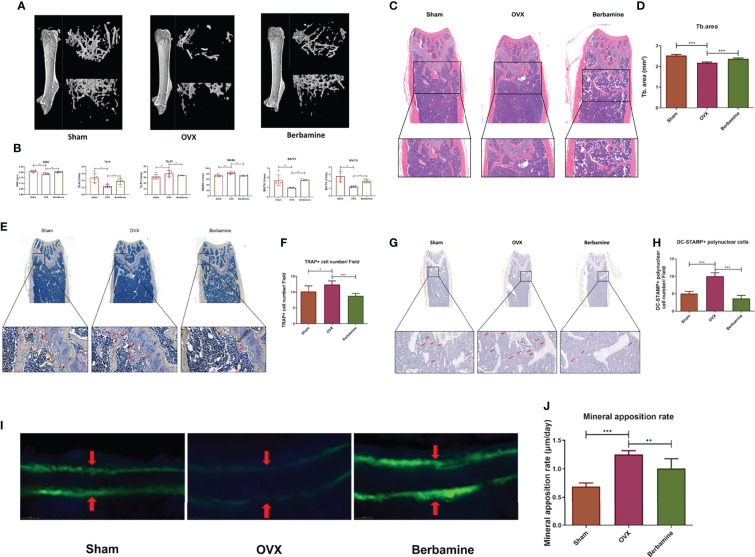
Berbamine can prevent bone loss by inhibiting osteoclast differentiation and osteoclast bone resorption function *in vivo*. **(A)** 3D reconstruction images of the distal femurs of mice in the sham, OVX and berbamine groups are presented. **(B)** The BMD, Tb.N, Tb.Pf, BS/BV, BS/TV, and BV/TV values of the distal femurs of the mice in the sham, OVX and berbamine groups are presented. **(C)** The hematoxylin-eosin staining of bone trabeculae in the metaphyses of the distal femurs of the mice in each group was quantified via ImageJ. **(D)** Quantification of the area of bone trabecula in distal femurs of mice in three groups, respectively. **(E)** TRAP immunohistochemical staining of distal femurs of mice in three groups, respectively, the red arrows point to TRAP-positive cells in the metaphysis of the distal femur. **(F)** Quantification of TRAP-positive cells in the metaphyses of the distal femurs of the mice in the three groups. **(G)** DC-STAMP immunohistochemical staining of distal femurs of mice in three groups, respectively, the red arrows point to DC-STAMP-positive cells in the metaphysis of the distal femur. **(H)** Quantification of DC-STAMP-positive cells in the metaphyses of the distal femurs of the mice in the three groups. **(I)** Calcein double labeling for measurement of femoral bone formation in mice in the sham, OVX, and berbamine groups. **(J)** Quantification of the MARs in the femurs of the mice in the sham, OVX and berbamine groups. The data are presented as the mean ± SD, *P < 0.05; **P < 0.01; ***P < 0.001.

According to the above results, we speculated that berbamine can alleviate the extent of PMOP by inhibiting osteoclast differentiation and osteoclast bone resorption function *in vivo*. Thus, we established a PMOP animal model *via* OVX. Berbamine (20 mg/kg) was administered by intraperitoneal injection. We used microcomputed tomography to scan the distal femurs of the mice, and we reconstructed 2D and 3D images of the distal femurs. Related parameters were analyzed, including the BMD, BV/TV, BS/TV, Tb.N and Tb.Pf. The extent of bone loss was significantly greater in the OVX group than in the sham group and the berbamine group, and there were significant differences in the parameters between the mice in the OVX group and those in the berbamine group (**Figures 5A, B**).

The results of hematoxylin-eosin staining of the distal femurs of the mice showed that the bone trabeculae were significantly more abundant in the berbamine group than in the OVX group (**Figures 5C, D**). In addition, there were significantly fewer TRAP-positive multinuclear cells in the distal femurs of the mice in the berbamine group than in the OVX group (**Figures 5E, F**). The results of DC-STAMP immunohistochemical staining showed that there were significantly fewer DC-STAMP-positive multinuclear cells in the distal femurs of mice in the berbamine group than in the OVX group (**Figures 5G, H**). The results of calcein double labeling for measurement of femoral bone formation showed that the MAR was significantly higher in the OVX group than in both the sham group and the berbamine group (**Figures 5I, J**).

## Discussion

Estrogen withdrawal often occurs in menopausal women and elderly women and can increase the formation and activation of osteoclasts (19, 20). Thus, inhibiting osteoclast formation and activation is an effective way to treat PMOP. For example, bisphosphonate has been widely used to treat PMOP because it inhibits osteoclast activation and osteoclast bone resorption function (19). However, bisphosphonate has side effects that cannot be ignored, such as atrial fibrillation and jaw osteonecrosis (21, 22). Thus, it is important to identify new drugs that can inhibit osteoclast activation and function effectively without significant side effects.

Berbamine is a natural bis-benzylisoquinoline that can be extracted from *Berberis amurensis* (23, 24), and has been widely used clinically in China for the treatment of leukopenia with various causes, especially chemotherapy (25). Several related studies have reported that berbamine has several pharmacological functions, such as anti-coronavirus (26), anti-inflammation (27), promoting tumor apoptosis (28) and immunomodulatory (29) functions. For example, previous research has reported that berbamine can suppress triple-negative breast cancer *via* the PI3K/Akt/MDM2/p53 and PI3K/Akt/mTOR signaling pathways (30). Berbamine can also inhibit the secretion of inflammatory mediators from macrophages and neutrophils *via* inhibition of the NF-κB and MAPK signaling pathways (18). It has been reported that the PI3K/Akt/mTOR, NF-κB and MAPK signaling pathways are closely related to osteoclastogenesis (31, 32). However, the inhibitory effect of berbamine on osteoclastogenesis and its mechanism have not been studied. Thus, we carried out this study to explore whether berbamine can ameliorate PMOP.

In this study, we revealed that berbamine can attenuate PMOP by inhibiting osteoclastogenesis of BMMs *in vitro* and *in vivo*. *In vitro*, the results showed that berbamine significantly inhibited RANKL-induced osteoclastogenesis of BMMs. Berbamine significantly suppressed the expression of osteoclast-related genes, including NFATc1, CD44, CTSK, MMP-9 and DC-STAMP, and downregulated the expression of the corresponding proteins. Notably, fusion of osteoclast precursors is one of the final steps of osteoclast formation, along with activation of bone resorption (33). Thus, failure of osteoclast fusion can seriously affect the bone resorption function of osteoclasts (34). We found that the inhibitory effect of berbamine on osteoclastogenesis of BMMs mainly occurred in the middle and late phases *in vitro*, which influenced the fusion of osteoclast precursors by inhibiting the mRNA and protein expression of DC-STAMP and CD44. DC-STAMP and CD44 are very important for osteoclast precursor fusion. Previous related research has revealed that DC-STAMP-deficient cells cannot fuse to form mature osteoclasts (35), which can lead to bone resorption function inhibition. CD44 is a transmembrane glycoprotein that is expressed abundantly in osteoclast precursors (36). The related studies have demonstrated that RANKL stimulation can cause the internalization of surface DC-STAMP, only 45% of the original level of DC-STAMP of BMMs could be observed after 3 days of RANKL culture (37, 38), and this phenomenon mainly occurs in osteoclast precursors in the middle and late phases of osteoclastogenesis (38). Ya-Hui Chiu found an immunoreceptor tyrosine-based inhibitory motif (ITIM) in the intracellular tail of DC-STAMP (39). In addition, Ya-Hui Chiu discovered that ITIM-deleted DC-STAMP can inhibit the nuclear translocation of NFATc1 and that the gene and protein expression levels of NFATc1 can be downregulated significantly when the DC-STAMP gene is knocked out (40). NFATc1 is an important transcription factor in the process of osteoclastogenesis and has been proven to upregulate the expression levels of CTSK, CD44 and MMP-9 (41–43). Ryotaro Iwasaki observed TRAP-positive mononuclear osteoclasts in mice with DC-STAMP deficiency (44). Thus, we predict that berbamine can bind with DC-STAMP on the surfaces of osteoclast precursors in the middle and late phases of osteoclastogenesis, which can consequently inhibit the internalization of DC-STAMP. Then, the gene and protein expression of NFATc1 and the nuclear translocation of NFATc1 are also inhibited, which affects the gene and protein expression levels of MMP-9, CD44 and CTSK but not TRAP. We did not find that berbamine could inhibit RANKL-induced activation of the MAPK signaling pathway, NF-κB signaling pathway or PI3K-AKT-NFATc1 signaling pathway in the early phase of osteoclastogenesis, which was in accordance with the results of TRAP staining and podosome belt staining *in vitro*.

For our *in vivo* experiments, we treated PMOP mice with berbamine for 8 weeks. Micro-CT scanning, hematoxylin-eosin staining, DC-STAMP immunohistochemical staining and TRAP immunohistochemical staining of the distal femurs of the mice revealed that PMOP was significantly alleviated in the treated mice. Estrogen withdrawal results in increased bone resorption accompanied by an increase in global bone formation, and menopause can induce increases (37-52%) in bone formation markers (45). A related study has shown that MAR is significantly higher in OVX monkeys than in sham-operated monkeys (46). In the present study, we also found that the MARs of the OVX group was significantly higher than the MARs of both the sham group and the berbamine group. The bone loss of mice in berbamine group was significantly lower than that of mice in the OVX group, although the MAR of the OVX group was significantly higher than that of the berbamine group. These findings suggest that berbamine has an ameliorative effect on PMOP by inhibiting bone resorption of osteoclasts.

Osteoblasts play an important role in the progression of bone mineralization. Thus, we treated bone marrow mesenchymal stem cells (BMSCs) collected from six-week-old C57BL/6 mice with berbamine (6.25 μM). According to the results of alkaline phosphatase (ALP) activity staining of the BMSCs, we infer that berbamine does not significantly inhibit the differentiation and mineralization of osteoblasts in the concentration range of 0-6.25 μM (**Figure S2**).

There are several limitations of the present study that cannot be ignored. First, we explored the ameliorative effect of berbamine only on a PMOP animal model *in vivo* and *in vitro*. The effects of berbamine on other kinds of bone metabolic diseases, such as secondary osteoporosis and aseptic loosening, were not explored. Second, we did not detect the concentrations of osteogenesis-related biomarkers such as osteocalcin (OCN), osteoprotegrin (OPG) and runt-related transcription factor 2 (Runx2) in the serum of the mice. Finally, many drugs have chemical structures similar to that of berbamine, and their therapeutic effects need to be clarified in future work.

In summary, our study reveals that berbamine could be a potential therapeutic alternative for PMOP prevention and treatment.

## Data Availability Statement

The raw data supporting the conclusions of this article will be made available by the authors, without undue reservation.

## Ethics Statement

The animal study was reviewed and approved by The Animal Ethics Committee of Fudan University Pudong Medical Center.

## Author Contributions

CZ, ZZ, ZQ, and BY: conceptualization. CZ, ZZ, ZQ, and WS: methodology, data collection and data curation. CZ, ZZ, ZQ, Marjan Mohamadali, and Farnaz Ghorbani: data analysis and data interpretation. CZ and ZZ: drafting manuscript. ZQ, Behafarid Ghalandari and Shiva Irani: revising manuscript content. BY, CY, and ZQ: supervision, project administration and funding acquisition. All authors contributed to the article and approved the submitted version.

## Funding

This work was supported by the National Natural Science Foundation of China (82170897) and Outstanding Clinical Discipline Project of Shanghai Pudong (No. PWYgy2021-08).

## Conflict of Interest

The authors declare that the research was conducted in the absence of any commercial or financial relationships that could be construed as a potential conflict of interest.

## Publisher’s Note

All claims expressed in this article are solely those of the authors and do not necessarily represent those of their affiliated organizations, or those of the publisher, the editors and the reviewers. Any product that may be evaluated in this article, or claim that may be made by its manufacturer, is not guaranteed or endorsed by the publisher.
