# Supplementary figures and images for: The Dibenzyl Isoquinoline Alkaloid Berbamine Ameliorates Osteoporosis by Inhibiting Bone Resorption
Source: Front Endocrinol (Lausanne). 2022 May 18;13:885507. doi: 10.3389/fendo.2022.885507 (PMC9159364; doi:10.3389/fendo.2022.885507)

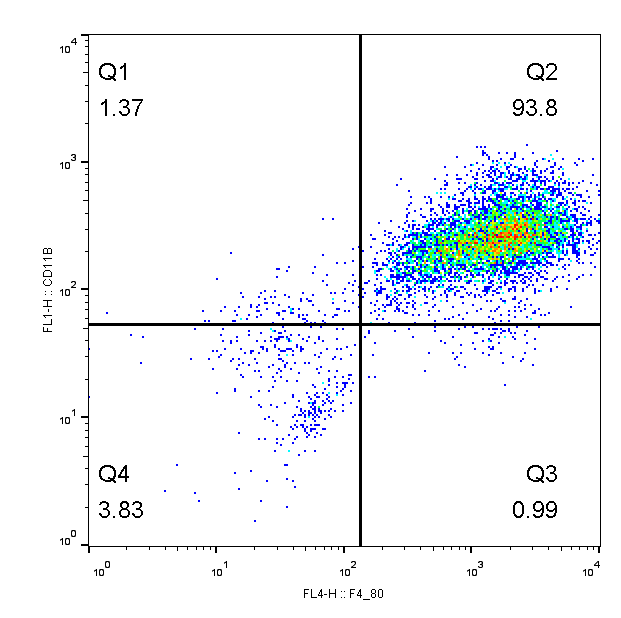

Supplement: Supplementary file 3 [file Image_3.png]
