# Supplementary material for: The Dibenzyl Isoquinoline Alkaloid Berbamine Ameliorates Osteoporosis by Inhibiting Bone Resorption
Source: Front Endocrinol (Lausanne). 2022 May 18;13:885507. doi: 10.3389/fendo.2022.885507 (PMC9159364; doi:10.3389/fendo.2022.885507)
Supplement: Supplementary file 4 [file Table_1.docx]

PMOP: postmenopausal osteoporosis

BMMs: bone marrow-derived macrophages

RANKL: receptor activator of nuclear factor-kappa B ligand

M-CSF: macrophage colony stimulating factor

MMP-9: matrix metalloproteinase 9

DC-STAMP: dendritic cell specific transmembarne protein

CTSK: cathepsin K

TRAP: tartrate-resistant acid phosphatase

RANK: receptor activator of nuclear factor-kappa B

TCM: traditional Chinese medicine

DMSO: dimethyl sulfoxide

CCK-8: Cell Counting Kit-8

α-MEM: alpha-modified minimal essential medium

P/S: penicillin–streptomycin

FBS: fetal bovine serum

IC50: half-maximal inhibitory concentration

qRT-PCR: quantitative real-time polymerase chain reaction

NFATc1: nuclear factor of activated T cells 1

SDS–PAGE: sodium dodecyl sulfate–polyacrylamide gel electrophoresis

PVDF: polyvinylidene fluoride

ECL: enhanced chemiluminescence

SPF: specific pathogen-free

OVX: ovariectomy

BMD: bone mineral density

Tb.N: trabecular number

Tb.Pf: trabecular pattern factor

BS/BV: bone surface /bone volume ratio

BS/TV: BS density /total volume

BV/TV: bone volume ratio/ total volume

MAR: mineral apposition rate

BMSCs: bone marrow mesenchymal stem cells

ALP: alkaline phosphatase

OCN: osteocalcin

OPG: osteoprotegrin

Runx-2: runt-related transcription factor 2
